# Cerebellar mutism syndrome: From pathophysiology to rehabilitation

**DOI:** 10.3389/fcell.2022.1082947

**Published:** 2022-12-02

**Authors:** Francesco Fabozzi, Stella Margoni, Bianca Andreozzi, Maria Simona Musci, Giada Del Baldo, Luigi Boccuto, Angela Mastronuzzi, Andrea Carai

**Affiliations:** ^1^ Department of Hematology/Oncology, Cell and Gene Therapy, Bambino Gesù Children’s Hospital, IRCCS, Rome, Italy; ^2^ Department of Pediatrics, Università degli Studi di Roma Tor Vergata, Rome, Italy; ^3^ School of Medicine, Sapienza Università di Roma, Rome, Italy; ^4^ School of Nursing, College of Behavioral, Social and Health Science, Clemson University, Clemson, SC, United States; ^5^ Faculty of Medicine and Surgery, Saint Camillus International University of Health Sciences, Rome, Italy; ^6^ Department of Neurosciences, Neurosurgery Unit, Bambino Gesù Children’s Hospital, IRCCS, Rome, Italy

**Keywords:** cerebellar mutism syndrome, posterior fossa, language, cerebellum, pediatric tumor

## Abstract

Cerebellar mutism syndrome (CMS) is a common complication following surgical resection of childhood tumors arising in the posterior fossa. Alteration of linguistic production, up to muteness and emotional lability, generally reported at least 24 h after the intervention, is the hallmark of post-operative CMS. Other associated traits include hypotonia and other cerebellar motor signs, cerebellar cognitive-affective syndrome, motor deficits from the involvement of the long pathways, and cranial neuropathies. Recovery usually takes 6 months, but most children are burdened with long-term residual deficits. The pathogenic mechanism is likely due to the damage occurring to the proximal efferent cerebellar pathway, including the dentate nucleus, the superior cerebellar peduncle, and its decussation in the mesencephalic tegmentum. Proven risk factors include brain stem invasion, diagnosis of medulloblastoma, midline localization, tumor size, invasion of the fourth ventricle, invasion of the superior cerebellar peduncle, left-handedness, and incision of the vermis. Currently, rehabilitation is the cornerstone of the treatment of patients with cerebellar mutism syndrome, and it must consider the three main impaired domains, namely speech, cognition/behavior, and movement.

## 1 Introduction

Central nervous system (CNS) tumors are the most common solid neoplasm occurring in children. They often arise in the posterior cranial fossa, and clinical presentation may include sign and symptoms of increased intracranial pressure, such as morning headache and vomiting, especially when an aggressive disease as medulloblastoma and ependymoma compress the fourth ventricle; conversely, less aggressive tumors as pilocytic astrocytoma usually present first with vague symptoms and then with ataxia of long duration. Overall, they almost invariably benefit from surgery as first-line treatment (Cohen, 2022). The extent of surgical resection represents an extremely significant prognostic factor in most CNS malignancies, leading to the introduction of the concept of maximal safe resection, the most extensive resection obtainable with a reasonable containment of functional risk. The recent availability of a complex technological toolkit to support the surgical resection of neoplasms, such as advanced navigation and visualization systems, intraoperative neuroimaging solutions as well as intraoperative monitoring techniques, has allowed a significant increase in the extent of resection with satisfactory functional results (Zebian et al., 2017).

Nevertheless, about a quarter of patients who undergo resection of posterior cranial fossa neoplasms experience a serious complication known as cerebellar mutism syndrome (CMS). Post-operative CMS was first described in 1939 and then more widely redefined starting from the mid-1980s based on experiences related to craniotomy for infratentorial brain tumors, with an incidence in this case even greater than 25% (Bailey et al., 1939; Rekate et al., 1985).

Post-operative CMS is characterized by an alteration of linguistic production, up to muteness and emotional lability, both generally reported at least 24 h after the intervention. Sometimes, hypotonia and other cerebellar motor signs, cerebellar cognitive-affective syndrome (CCAS), motor deficits from the involvement of the long pathways, and cranial neuropathies may occur in association. Recovery usually takes 6 months, but most children will suffer from long-term residual deficits.

In this review, we aim to summarize the current knowledge regarding the pathophysiology of CMS, to provide an overview of clinical manifestations, risk factors, and treatment, focusing on rehabilitation therapies.

## 2 The role of cerebellum in language

The cerebellum plays a crucial role in the control and regulation of motor functions: while it does not initiate movement, it contributes to its coordination, precision, and accurate timing, due to complex integrated systems. However, it is currently estimated that only 20% of cerebellar functions are related to motion and that the cerebellum is also involved in cognitive functions, such as attention, memory, and language, as well as in the regulation of responses to fear or pleasure, according to the theories of dysmetria of thought and the universal cerebellar transform. They argue that the cerebellum maintains behavior around a homeostatic baseline, without conscious awareness, driven by implicit learning, and performed according to context (Schmahmann, 2019; Schmahmann et al., 2019). Further confirmations of the dual nature (cognitive/emotional) of the cerebellum come from investigations performed by resting-state functional magnetic resonance (rsfMRI) imaging, conducted by requiring the performance of specific tasks: the motor networks of cerebral origin map on sensorimotor areas of the cerebellum, located in the anterior lobe and lobule VIII, whereas the networks underlying cognitive functions map to focal areas located in the posterior lobe of the cerebellum. More specifically, speech and verbal working memory activate lobules VI and Crus I; visuospatial tasks involve lobule VI; executive functions, such as working memory, planning, organization, and strategy development, activate lobules VI, Crus I, and VIIB; emotional responses involve lobules VI and VII; finally, hemispherical lobules VI, Crus I, and VIIB also appear to be involved in encoding generalized aversive processing (Stoodley and Schmahmann, 2018; Schmahmann, 2019). Available data suggest lateralization of function, with language representation being more often reported on the right cerebellum and spatial functions to the left, reflecting the crossed cerebro-cerebellar projections (Klein et al., 2016). The production of sounds typical of spoken language is a complex process that is believed to involve mechanisms such as lexical selection, phonological coding, phonetic planning, and articulation, thus constituting a competence that cannot completely be separated from other cognitive functions, the integrity of which it also depends on cerebellar coordination. The theory is that the meaning and sounds of words are retrieved from a mental dictionary (lexical selection) and mapped into the rhythm of the word (phonological coding), planned for motor processing of the individual syllables (phonetic planning) and implemented as a coordinated motor output specific to the joint. While the cerebellum is believed to be engaged in lexical selection, its role has not been considered significant in studies investigating phonological coding and phonetic planning. As the coordinator of motor functions, it also predictably plays an important role in articulation, where speech sounds are produced; however, even hidden articulation such as silent counting and syllable repetition seems to depend on it (Castelli, 2020). Thus, the cerebellum, considered for a long time a complex associative center related to the processing of motor and executive functions, is currently recognized as an associative area also responsible for the processing of higher mental, cognitive and emotional functions, from a very early age (Koziol et al., 2014).

## 3 Pathophysiology of CMS

Since Since CMS is mostly found in pediatric patients who undergo surgery for a cerebellar tumor, much of the current knowledge regarding the pathogenetic mechanisms comes from studies of children with medulloblastoma (Catsman-Berrevoets and Patay, 2018).

Post-operative CMS is currently believed to occur due to the surgical injury of anatomical structures that connect the cerebellum to the brainstem: more specifically, there is increasing evidence from data collected based on advanced MRI studies such as diffusion sequences and tractography that damage to the proximal efferent cerebellar pathway (ECP) is likely to be considered as the anatomical substrate of post-operative CMS. The proximal ECP includes the dentate nucleus, the superior cerebellar peduncle, and its decussation in the mesencephalic tegmentum, while its fibers travel towards the red nucleus and the thalamus (dentato-rubro-thalamic tract, DRTT) (Grønbæk et al., 2021). In particular, significant results come from a study conducted on 28 children with medulloblastoma who underwent resective surgery (Wells et al., 2010): 11 (39%) of these children then developed CMS. Images obtained immediately after surgery showed cerebellar edema in 92% of all patients, with a greater tendency to localization in the middle and upper cerebellar peduncle in patients with CMS (*p* = 0.05 and 0.07, respectively). Finally, further confirmation of the involvement of cerebellar peduncles comes from a more recently published retrospective cohort study (Toescu et al., 2018a), conducted by enrolling 56 children diagnosed with medulloblastoma. Of these, 12 (21.4%) developed post-operative CMS. In scans conducted early (median 5 days) and then later (median 31 months) after resection, T2-weighted change in superior cerebellar peduncle was more common in the group of patients diagnosed with post-operative CMS (*p* = 0.040 and 0.046 respectively), flanked by a statistically significant signal alteration in the dentate nuclei (*p* = 0.024). These results confirm the crucial etiopathological role played by lesions to the ECP in the context of post-operative CMS (Toescu et al., 2018a).

However, beyond this evidence, there is still no unifying and shared etiological hypothesis that clarifies in detail the pathophysiology of this condition. One of the suggested mechanisms is cerebral-cerebellar diaschisis (Miller et al., 2010). The term diaschisis is defined as a sudden suspension or inhibition of function in one area of the brain after damage to another distant region that provides input to the former. Thus, a DRTT lesion causes loss of excitatory impulses from the cerebellum to areas of the cerebral cortex such as the motor, premotor, and prefrontal regions, resulting in their loss of function; all these areas are involved in both motor and cognitive functions impaired in CMS (Miller et al., 2010). Hypoperfusion, reduced oxygen consumption, and hypometabolism of the cerebral cortex due to the lack of cerebellar input are the hallmarks of cerebral cerebellar diaschisis, as found in studies by Miller and Catsman-Berrevoets (Catsman-Berrevoets and Aarsen, 2010; Miller et al., 2010). Diaschisis is a dynamic process that may get better over time, explaining the characteristic improvement of CMS (Catsman-Berrevoets and Patay, 2018). However, recent studies have also shown that parenchyma affected by diaschisis may suffer from long-term damage (McEvoy et al., 2016), which may underlie the language and cognitive deficits observed in a significant number of patients recovered by CMS (Robertson et al., 2006).

## 4 Clinical features of post-operative cerebellar mutism

CMS is characterized by the onset of mutism or severely poor speech associated with emotional lability after surgical resection (Tamburrini et al., 2015; Schmahmann, 2020; Ashida et al., 2021). Typically, the surgery is followed by an interval of time, even a few days, during which the child remains able to speak. However, the onset is usually early and occurs within the first 24–48 h.

Complete, albeit transient, speech loss then evolves into dysarthria, with the recovery of complex movements of the mouth and tongue preceding recovery from mutism (De Smet et al., 2007; Mourik et al., 2008). Indeed, the improvement of speech seems to be sensitive to rehabilitation techniques that involve the complex muscular movements of the tongue and mouth, although burdened by difficult recovery (van Dongen et al., 1994). Of note, the hallmark characteristics of ataxic dysarthria, such as irregular articulatory breakdown and scanning speech, are often not part of the clinical picture of post-mutism dysarthria (Steinbok et al., 2003), suggesting that a higher-level motor planning disorder (apraxia) may underlie the speech disorder (De Witte et al., 2017).

Hypotonia and ataxia are the most common accompanying symptoms, followed by cranial nerve deficit and brainstem station damage. Dysphagia is also very common, due to lesions of the truncal centers or cranial nerves. The post-operative CMS is also characterized by a broad spectrum of emotional and behavioral disorders that include states of profound irritability, communication disorders, and a tendency to isolate up to an autistic-like framework. These behavioral aspects are associated with agrammatic language disorders and verbal stereotypies (Riva and Giorgi, 2000).

Although recovery from mutism occurs in virtually all cases in a time interval usually ranging from a few days to 6 months, the constellation of dysfunctional deficits in the area of language, speech, and communication that are related to posterior cranial fossa tumors (especially at the cerebellar level), tend to improve but not disappear. Indeed, several studies have demonstrated an increased likelihood of long-term motor and non-motor neurological deficits as long as 5 years after tumor resection (Steinbok et al., 2003; Aarsen et al., 2004; Huber et al., 2006), showing that CMS represents a significant risk factor for cognitive deficits in pediatric CNS tumor survivors.

**FIGURE 1:**
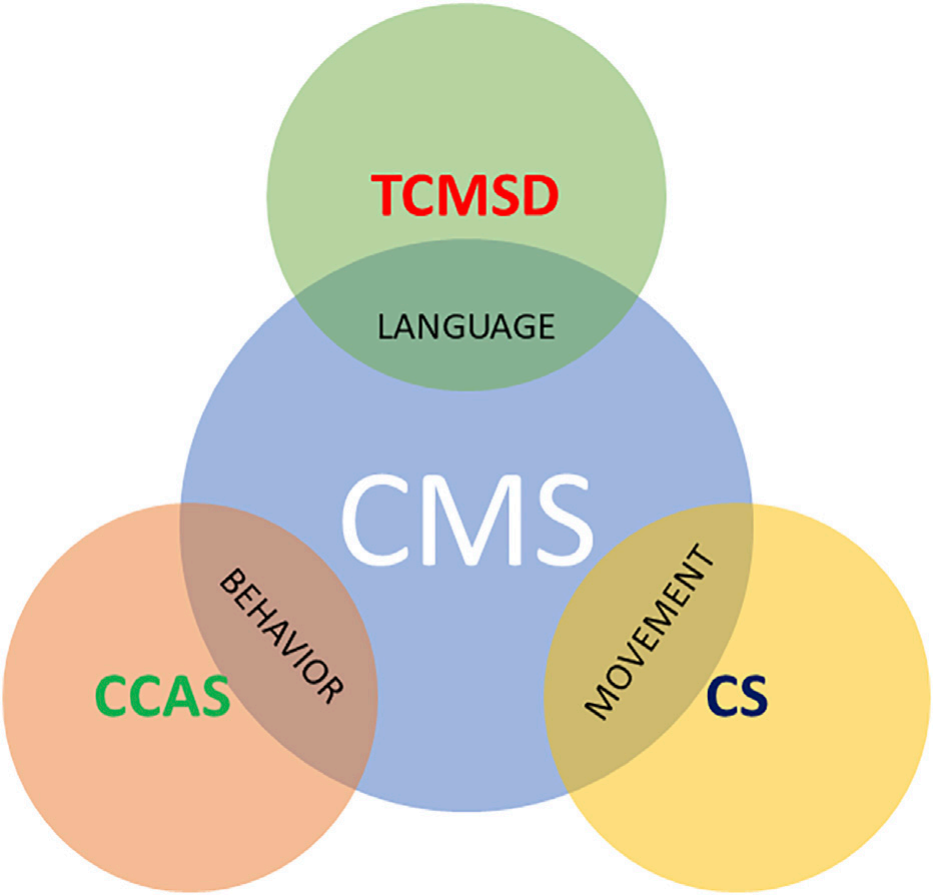
Some terms found in the literature to describe CMS. CCAS highlights behavioral changes, whereas TCMSD focuses on mutism and CS indicates the typical motoric cerebellar signs that can be observed both pre- and post-operatively.CMS, cerebellar mutism syndrome; CCAS, cerebellar cognitive-affective syndrome; TCMSD, transient mutism and subsequent dysarthria; CS, cerebellar syndrome.

Despite CMS having been known for several decades, there is still widespread inconsistency in the terminology and its definition. Several terms have been used in literature: cerebellar mutism, posterior fossa syndrome, CMS, cerebellar cognitive-affective syndrome (CCAS), transient cerebellar mutism and subsequent dysarthria, and akinetic mutism, with each of these definitions, focused on some aspects of the disease rather than others (Figure 1). Posterior fossa syndrome is made up of linguistic, neurobehavioral, and motoric components as well as mutism syndrome and subsequent dysarthria focuses on the linguistic part of the spectrum; on the other hand, the cerebellar syndrome is a synonym for typical motoric cerebellar signs that can be observed both pre- and post-operatively. The CCAS sheds light on the affective component, with manifestations such as irritability, impulsivity, disinhibition and lability of effects, and poor attentional and behavioral modulation, and was firstly described by Schmahmann in an adult population (Schmahmann and Sherman, 1998; Schmahmann, 2021) and subsequently recognized by Levisohn in children underwent surgical excision of medulloblastoma (Levisohn et al., 2000). Finally, CMS covers some of the most characteristic features of each category.

This wide variety of definitions not only reflects an academic debate, but also the difficulty in evaluating and quantifying this syndrome, characterized by marked heterogeneity in clinical manifestations and diagnostic findings (Gudrunardottir et al., 2011; Schmahmann, 2020). In May 2016, an international group of clinicians and researchers from different disciplines met to formulate a new definition of CMS and standardized methods for diagnosis and follow-up (Gudrunardottir et al., 2016). In the article produced by consensus, the term posterior fossa syndrome was retired, and post-operative CMS is now defined as a syndrome characterized by delayed onset mutism/reduced speech and emotional lability after posterior fossa tumor surgery in children. Hypotonia and oropharyngeal dysfunction/dysphagia are further common features. It may frequently be accompanied by cerebellar motor syndrome, CCAS, and brain stem dysfunction as long tract signs and cranial neuropathies. The mutism is always transient, but recovery from CMS may be prolonged. Speech and language may not return to normal as well as other deficits of cognitive, affective, and motor functions often persist (Gudrunardottir et al., 2016).

Further progress derives from the foundation of the Posterior Fossa Society, an association aimed at systematically collecting and exchanging information on CMS and CCAS. The future research objectives, towards which we must direct ourselves, as clarified in the article, “Posterior Fossa Society Consensus Meeting 2018: a synopsis” (Molinari et al., 2020), currently include:

1. Refine the definition and evaluation of CMS symptoms through scoring;
2. Understand the pathogenesis and improve risk stratification strategies;
3. Develop rehabilitation approaches and protocols.

The following thesis therefore arises in response to the unanimous desire to obtain further data-based information, promote and increase knowledge of CMS, formulate official guidelines and guide future research efforts (Tamburrini et al., 2015).

However, several issues still remain debating. Of note, a significant number of patients undergone surgical resection of medulloblastoma experience severe ataxia and/or behavior changes without speech alterations, as shown in the prospective study by Khan and colleagues (Khan et al., 2021). Based on these findings, in the future all these manifestations could encompass under an “umbrella” post-surgical syndrome, of which CMS representing the most severe spectrum.

## 5 Risk factors and risk stratification

The small sample size and the heterogeneity of many of the CMS studies have hindered the identification of significant risk factors over the years. Only recently two reviews have been published (Ashida et al., 2021; Pettersson et al., 2022) aimed at identifying statistically significant risk factors for the onset of post-operative CMS, aggregating data from more than 1,000 patients. The results are summarized in Table 1. Proven risk factors identified include brain stem invasion, diagnosis of medulloblastoma, midline localization, tumor size, invasion of the fourth ventricle, invasion of the superior cerebellar peduncle, left-handedness, and incision of the vermis.

**TABLE 1:** Risk factors based on (Ashida et al., 2021; Pettersson et al., 2022) finding are summarized.

Risk factor	OR (95% CI)	Statistical significance
Brain stem invasion	4.28 (2.23–8.23) (Pettersson et al., 2022)	*p* < 0.0001 (Pettersson et al., 2022)
	3.38 (2.22–5.15) (Ashida et al., 2021)	*p* < 0.00001 (Ashida et al., 2021)
Midline localization	1.75 (1.15–2.67) (Ashida et al., 2021)	*p* = 0.009 (Ashida et al., 2021)
Tumor size	8.85 (1.30–60.16) (Pettersson et al., 2022)	*p* = 0.03 (Pettersson et al., 2022)
	4.09 (1.63–9.93) (Ashida et al., 2021)	*p* = 0.002 (Ashida et al., 2021)
Invasion of the 4th ventricle	12.84 (4.29–38.44) (Ashida et al., 2021)	*p* < 0.00001 (Ashida et al., 2021)
Invasion of the SCP	6.77 (2.35–19.48) (Ashida et al., 2021)	*p* = 0.0004 (Ashida et al., 2021)
Left-handedness	6.57 (1.25–34.44) (Pettersson et al., 2022)	*p* = 0.03 (Pettersson et al., 2022)
Incision of the vermis	5.44 (2.09–14.16) (Pettersson et al., 2022)	*p* = 0.0005 (Pettersson et al., 2022)
	2.61 (1.54–4.43) (Ashida et al., 2021)	*p* < 0.00001 (Ashida et al., 2021)
Diagnosis of medulloblastoma	3.26 (1.93–5.52) (Pettersson et al., 2022)	*p* < 0.0001 (Pettersson et al., 2022)
	4.3 (2.60–7.12) (Ashida et al., 2021)	*p* < 0.00001 (Ashida et al., 2021)

OR, odds ratio; CI, confidence interval; SCP, superior cerebellar peduncle.

Brain stem invasion was found as a significant risk factor in both studies, confirming the results of Robertson and colleagues, who identified brain stem invasion as the only risk factor that correlated positively with the development of CMS (Robertson et al., 2006). Similarly, brain stem involvement was found in all patients in the study by Doxey et al. (Doxey et al., 1999). Midline localization was proven as a significant risk factor when compared with non-midline tumors, based on studies including a total of 528 patients (Ashida et al., 2021). Previously, a significant increase in the risk of developing CMS (*p* = 0.04) was reported by Kotil and co-workers in a small case series (Kotil et al., 2008). Tumor size > 5 cm was also found as a significant risk factor in both reviews. All these proven risk factors, also including the invasion of the fourth ventricle and the invasion of the superior cerebellar peduncle, are likely based on the same anatomical basis: the increased risk of damage to the DRRT pathways during surgery. Other risk factors are left-handedness and incision of the vermis compared with the telovelar approach. Overall, the diagnosis of medulloblastoma compared to other types of cancers has long been considered a known risk factor for post-operative CMS, and both the results by Petterson and Ashida have confirmed this. In addition to the high incidence in the pediatric setting, this is most likely related to the common location of the medulloblastoma along the midline and involvement of the brain stem, both of which are produced by unknown biological mechanisms. In recent years, four molecular subgroups of MB have been identified through transcriptional profile studies: sonic hedgehog [SHH], wingless [WNT], group 3, and group 4 (Ramaswamy and Taylor, 2017; Northcott et al., 2019). In this sense, it is interesting to note that several studies have recently identified the SHH subgroup as a significant factor in reducing the risk of post-operative CMS (Jabarkheel et al., 2020). The influence in the onset of post-operative CMS is probably the result of the cellular origin of a the subgroup rather than of the molecular characteristics: indeed, SHH medulloblastoma originates from the outer granular layer and therefore the tumor will typically develop within the cerebellar hemispheres; on the other hand, the origins from the lower rhombic lip, Nestin + cells, and unipolar brush cells, respectively of the WNT subgroups, 3 and 4 (Vladoiu et al., 2019), will give rise to tumors with an intimate relationship with the structures of the fourth ventricle, causing a greater risk of damage to the DRTT pathway during resection. However, despite the SHH subgroup having been reported as a risk-reducing factor for post-operative CMS, molecular stratification is rarely available at the time of surgical resection. Of note, a recent prospective multicenter cohort study further confirmed that a midline tumor location and high-grade tumor histology (namely, medulloblastoma and atypical teratoid rhabdoid tumor compared to astrocytoma) increase the risk of developing CMS, and also younger age was recognized as a risk factor; conversely, whereas the transvermian surgical approach compared with telovelar was not found as a risk factor (Grønbæk et al., 2021). This discrepancy can be partially explained by two factors: first, the telovelar approach requires greater technical skill, and is therefore practiced by experienced surgeons; and second, the transvermian approach is preferred in the presence of large tumors, a well-established risk factor, introducing an additional bias. In fact, in another prospective study, surgery in a low-volume surgery center were found to increase the likelihood of CMS (Khan et al., 2021), underscoring the role of surgeon ability. In this regard, the use of supportive techniques such as intraoperative imaging, a rapidly expanding technology in neurosurgery, capable of providing improved resection extension and surgical precision, thereby sparing healthy brain tissue, could reduce the risk of post-operative CMS, as shown by Petterson meta-analysis (OR 0.36, 95% CI 0.18–0.72*; p* = 0.004) (Khan et al., 2021; Pettersson et al., 2022).

### 5.1 Risk stratification

In recent years, several models of preoperative risk stratification have been investigated, using both imaging and clinical features (Liu et al., 2018; Zhang et al., 2019; Bae et al., 2020). The estimation of a high risk of mutism following a complete resection has implications not only to the extent that this must be carefully discussed by the neurosurgical team but also for the surgical strategy itself. Detailed preoperative imaging and technical advances have prompted neurosurgeons in recent years to increasingly attempt complete resection. Similarly, there have been substantial advances in understanding the biology of pediatric brain tumors, such that a considerable number of patients can be cured even when there is a residual gross tumor left after resection. Multidisciplinary neuro-oncology teams will recognize the potential benefit of having a patient who is clinically in good condition immediately after surgery and able to proceed rapidly to adjuvant therapy versus one who has undergone a complete resection but is unable to proceed to prompt subsequent radiotherapy due to the profound physical, cognitive and communication difficulties inherent in the post-operative CMS. Furthermore, the potentially permanent consequences of cognitive, physical, and communication problems can be a high price to pay for treatment. Patients at higher risk with favorable medulloblastoma histology might benefit in the future from intentional submaximal resection or a 2-step surgical strategy with debulking chemotherapy. Increasingly, discussions among pediatric neurosurgeons suggest we may be on the verge of an era of less aggressive surgery for selected patients. In addition to having a potential impact on the assessment process and surgical strategy, a risk score for the development of post-operative CMS in the future could also be useful in selecting patients for preventive neuroprotective therapies once these have been developed (Toescu et al., 2018b; Liu et al., 2018; Bae et al., 2020).

## 6 Management of postoperative speech impairment

CMS and associated symptoms often affect the healthy social development of affected children. Long-term multimodal rehabilitation is therefore often needed, with an emphasis on family involvement.

At present, no treatment protocol has been established and the effects of pharmacological interventions are sporadically reported in the literature (Noris et al., 2021) (Table 2). Likewise, it is not known whether routine administration of corticosteroids to patients with brain tumors affects the overall clinical course of CMS positively or negatively (Catsman-Berrevoets, 2017; Catsman-Berrevoets and Patay, 2018). While preoperative corticosteroids are routinely given to most patients, intra and post-operative administrations vary from center to center. There are no current neurosurgical recommendations regarding the post-operative use of corticosteroids, but they are often administered to reduce surgery-induced edema.

**TABLE 2:** Medical treatments reported in literature are summarized.

Treatment	Number of cases	References
Fluoxetine	3	(Akhaddar et al., 2012; Amor-García et al., 2021)
Bromocriptine	9	(Echiverri et al., 1988; Caner et al., 1999; Adachi et al., 2005; Amor-García et al., 2021)
Zolpidem	1	Shyu et al. (2011)
Midazolam	1	Nicita et al. (2017)
Haloperidol, delorazepam, risperidone	1	Noris et al. (2021)

Bromocriptine, fluoxetine, haloperidol, risperidone, delorazepam, and zolpidem have been widely evaluated as potential treatments; however, all these studies involved too small samples to show relevant results (Echiverri et al., 1988; Black and Uhde, 1994; Dummit et al., 1996; Caner et al., 1999; Adachi et al., 2005; Gudrunardottir et al., 2011; Shyu et al., 2011; Akhaddar et al., 2012; Noris et al., 2021). The beneficial effect of all these drugs appears at least 24 h after the first administration and usually, complete recovery takes some months, making it difficult to assess the real contribution of therapy to healing. On the other hand, Nicita and colleagues reported a resolution of post-operative CMS within a few minutes after intravenous administration of midazolam in a 17-year-old boy who had undergone a fourth-ventricle choroid plexus papilloma resection (Nicita et al., 2017).

### 6.1 Rehabilitation issues

**FIGURE 2:**
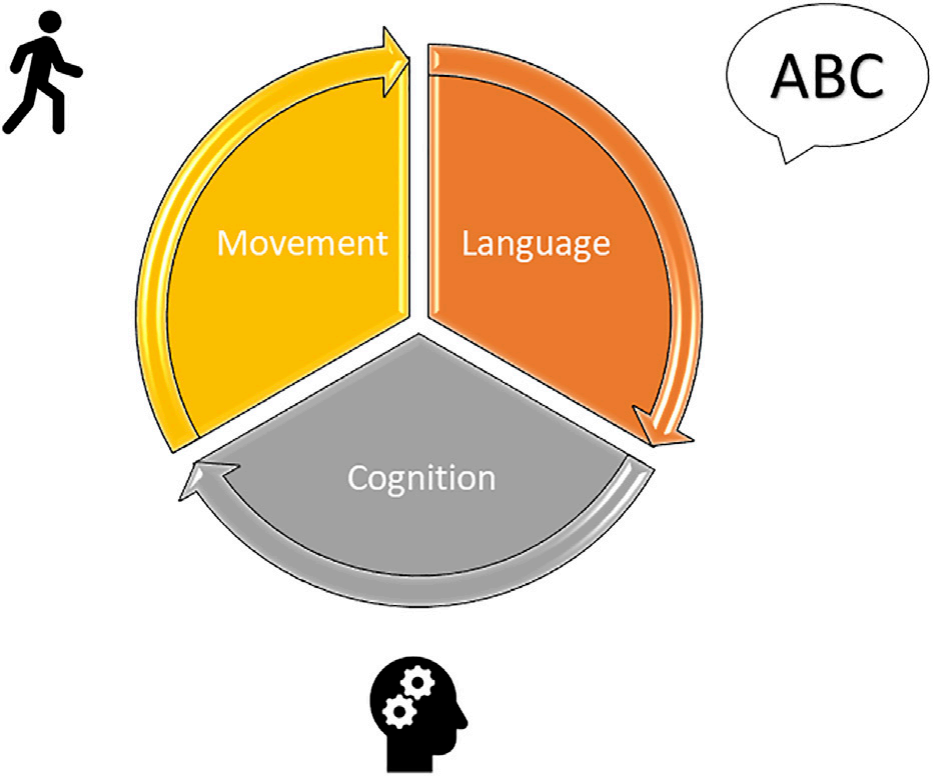
Rehabilitation of pediatric patients with CMS must consider the three main impaired domains, namely speech, cognition/behavior, and movement.

The significant role of therapy rehabilitation is that it must always be provided for by an adequate therapeutic plan, and of which after pre- and post-operative evaluations, aims to set up a long-term follow-up. Acquiring information on the site of the lesion, on the medical and therapeutic path undertaken and/or in progress, and considering the possible symptoms that may be encountered are necessary actions for the therapists to orient themselves correctly during this phase. Based on the framework, the possible short, medium, and long-term objectives will be indicated, which consider the priorities for the young patient. Each goal should support the child in social and school reintegration, also considering the evolution over time of the cognitive and linguistic profile and the possible future difficulties that the child will encounter. The treatment should be carried out in a context that is as ecological as possible; the techniques used are many and linked to the experience of the individual therapist since there is no evidence in the literature of more or less valid treatments for children with a brain tumor in the posterior cranial fossa (Castelli, 2020; Paquier et al., 2020). Overall, the therapy evaluation and the rehabilitation approach of pediatric patients with brain tumors require consideration of multiple aspects inherent to the disease itself, considering the three main impaired domains, namely speech, cognition/behavior, and movement (Figure 2) (Paquier et al., 2020). Thus, a collaboration among several professionals, namely a physiotherapist, a speech therapist, specifically a deglutologist, a neuro- and psychomotor therapist, a psychologist, and a neuropsychiatrist, must be warranted.

#### 6.1.1 Speech rehabilitation

The setting up of a speech therapy rehabilitation program in the post-operative setting is a fundamental part of the therapy and management of the clinical picture of CMS and is modulated on the specific deficits shown by each patient. Since an early intervention in children treated for brain tumors has been reported to effectively minimize speech and language deficits, a prompt evaluation should be recommended (Skinner et al., 2015).

The primary objective of the speech evaluation will be to identify the preserved functions, those deficient, and those not present (Castelli, 2020). In this setting, an important factor to consider is the age of the patient. Early injuries, especially if occurring before the age of six, are known to have greater repercussions of brain damage in later development (Adducci and Poggi, 2011; Day et al., 2021). Therefore, the damage from non-acquisition or non-exposure suffered by a child with a particular neurological condition should always be taken in account.

Firstly, it may be useful to investigate the previous language skills and possibly the child’s academic level. To this end, the conversation with the parents on the pre-morbid condition of the young patient, and when possible, the viewing of films before treatment is crucial. In addition to questions about language development, if the child is of school age, it may be helpful to look at notebooks from previous years. Whenever possible, the interview with the child’s teachers can also provide useful information (Walker et al., 2014; Paquier et al., 2020). A first observation can be made by suggesting a game or activity suitable for the child’s age that does not overly press on their difficulties. The observation phase cannot be limited to a single session but will require several meetings. Also, it must be considered that in the first few weeks following surgery and adjuvant treatments, the child is in a relatively rapid transition phase; therefore, subjecting them to a battery of standardized and structured tests can be extremely tiring for the patient, making the attempt to outline a realistic picture of his competences unsuccessful. This reflection is even more valid in severe cases, such as children with post-operative CMS (Paquier et al., 2020).

By comparing the pre- and post-operative evaluations, the type of linguistic disability reported by patients can be defined, directing them toward the most suitable rehabilitation program. As supported by the relevant literature, two profiles with different neurocognitive characteristics have been differentiated (Tamburrini et al., 2015):

1. Pure dysarthria, within a framework of adequate neurocognitive functions and linguistic understanding. In this profile there are involuntary movements of the tongue and lips and the inability to imitate buccolingual praxis upon request;
2. Apraxia of speech with transient dysmetria of thought that involves praxis and procedural memory disorders. In this profile, the language is telegraphic, consisting mainly of single nouns or verbs conjugated to the infinitive, devoid of grammatical and syntactic elements. The articulation is not dysarthria, but slow and monotonous, devoid of prosody.

Such a distinction allows speech therapists to orient themselves more easily in the implementation of a rehabilitation plan. If the child has a pure dysarthria disorder, rehabilitation is suggested that aims to support better pneumo-phono-articulatory coordination, better sensor-motor reintegration, and training in reorganizing the output word patterns. If the child presents the second profile, the objective of the rehabilitation program is to strengthen phonological awareness, the representation of sounds, and the planning of their sequences. Beyond the type of alteration reported, which is not always exactly definable, the various skills to be rehabilitated are the following:

1. Breathing and phonation. The overall goal of this first rehabilitation field is to harmonize the pneumo-phono-articulatory coordination, stimulated by referring to the necessary coupling between the expiratory and articulatory phases, which must meet according to a correct timing that must be studied together with the subject;
2. Improvement of speech (word articulation): exercises of repetition, reading, and description with or without an iconic stimulus are combined with elements taken from the PROMPT (Prompts for Restructuring Oral Muscular Phonetic Targets) method, an approach used to develop the motor skills involved in language. Tactile inputs are therefore provided to the organs affected by articulation, such as the jaw, labio-facial muscles, and tongue, incorporating these stimuli with visual and auditory information;
3. Strengthening of the lexical and semantic area through an expansion of the semantic network and of the access skills to lexical labels;
4. Pragmatics: pragmatics deals with exploring how the context affects the interpretation of meanings, thus evaluating whether the child is assertive, responsive, and respectful of ‘conversational maxims’, the regulating principles that govern conversation according to logic and relevance, respecting the cooperation between speakers;
5. Enhancement of cognitive and cognitive-linguistic functions: execution of exercises aimed at strengthening attention, memory, working memory, planning and problem-solving skills, relationships/associations, abstract reasoning, categorization, and inferences.

#### 6.1.2 Cognitive/behavioral rehabilitation

Cognitive rehabilitation programs are mainly carried out in both face-to-face therapeutic sessions and computer-based intervention, or with a combination of these two approaches (Walker et al., 2014; Paquier et al., 2020). Several functions such as executive function, attention, memory, and academic achievement should be targeted (Chieffo et al., 2022). Main remediation programs studies are summarized in Table 3.

**TABLE 3:** Main cognitive remediation programs are summarized. Two distinct approached are utilized: face-to-face therapeutic sessions, and computer-based intervention. None of these studies focuses on children affected by pCMS.

Remediation program	Main results	References
Face-to-face therapeutic sessions targeting: hierarchically graded massed practice; strategy acquisition; cognitive-behavioral therapy	Significant improvements in academic achievement, attention, and improved implementation of metacognitive skills	Butler et al. (2008)
Educational therapy and cognitive-behavioral approaches aiming to teach problem-solving skills and compensatory strategies designed to improve daily problem solving, attention and memory, and academic performance	Poor compliance with the treatment, raising concern about widespread acceptance of such programs in the oncology clinic	Patel et al. (2009)
Computerized cognitive training program (Captain’s Log)	Improvements in working memory and decreases in parent-rated attention problems	Hardy et al. (2011)
Computerized working memory training program (CogmedRM)	Improvements in visual working memory and in parent-rated learning problems	Hardy et al. (2013)
Computerized working memory training program (CogmedRM)	Strong compliance showed that Cogmed is a feasible and acceptable intervention	Cox et al. (2015)
Computerized working memory training program (CogmedRM)	Improvement on measures of working memory, attention, and processing speed	Conklin et al. (2015)

#### 6.1.3 Motor rehabilitation

Acute inpatient multidisciplinary rehabilitation is reported to improve motor outcomes in children with brain tumors (Philip et al., 1994). Yet, evidence regarding physical rehabilitation for children with CMS as well as the most beneficial type and intensity of rehabilitation programs are lacking (Paquier et al., 2020). However, conventional physical therapy represents an option to be considered, in keeping with results reported in children with ataxia following traumatic and acquired brain injury (Sartor-Glittenberg and Brickner, 2014).

### 6.2 Future directions

Nowadays, new technologies such as brain computer interfaces, robotics, virtual reality, and augmented reality tools have implemented rehabilitation strategies, allowing more oriented and more tailored treatments to be administered to the patient, also making rehabilitation possible at the patient’s home, with indisputable logistic and psycho-emotional advantages and bringing indisputable advantages also in terms of motivation in the post-operative rehabilitation of the patient with tumor of the posterior cranial fossa (Clemenson and Stark, 2015; Corbetta et al., 2015; Jung et al., 2020; Lino et al., 2021; Sciancalepore et al., 2022).

Furthermore, the most recent medical literature reports interesting data on the positive impact on clinical recovery of rehabilitation programs that include aerobic physical motor activity in pediatric neuro-oncological patients (Szulc-Lerch et al., 2018; Kohler et al., 2021).

Alternative procedures such as transcranial magnetic stimulation (TMS) and transcranial direct current stimulation—especially in combination with cognitive interventions—are promising techniques for stimulating neurologic recovery and plasticity in adults. TMS has been found to be safe in children and adolescents with CNS disorders. Exploring the effect of TMS alone or combined with a cognitive intervention in children with CMS may lead to new therapeutic opportunities (Allen et al., 2017; Sathappan et al., 2019).

## 7 Conclusion

CMS related to posterior cranial fossa tumors carries a constellation of dysfunctional deficits in the areas of language, behavior, and movement that tend to improve but not disappear. It is, therefore, necessary that the approach to the patient is multidisciplinary and timely, providing for an accurate assessment followed by an early and adequate rehabilitation treatment course by experts in the field.
